# Efficacy and safety of hormone therapies for treating adenomyosis-associated pelvic pain: a systematic review and network meta-analysis of randomized controlled trials

**DOI:** 10.3389/fendo.2025.1571727

**Published:** 2025-03-17

**Authors:** Andrea Etrusco, Vittorio Agrifoglio, Antonio D’Amato, Vito Chiantera, Antonio Simone Laganà, Joe Haydamous, Luigi Cobellis, Pasquale De Franciscis, Silvia Vannuccini, Harald Krentel, Antoine Naem, Gaetano Riemma

**Affiliations:** ^1^ Department of Health Promotion, Mother and Child Care, Internal Medicine and Medical Specialties (PROMISE), University of Palermo, Palermo, Italy; ^2^ Unit of Obstetrics and Gynecology, “Paolo Giaccone” Hospital, Palermo, Italy; ^3^ Department of Interdisciplinary Medicine (DIM), Unit of Obstetrics and Gynecology, University of Bari “Aldo Moro”, Policlinico of Bari, Bari, Italy; ^4^ Unit of Gynecologic Oncology, National Cancer Institute - IRCCS - Fondazione “G. Pascale”, Naples, Italy; ^5^ Department of Obstetrics Gynecology and Reproductive Sciences, McGovern Medical School at The University of Texas Health Science Center at Houston (UT Health), Houston, TX, United States; ^6^ Department of Woman, Child and General and Specialized Surgery, University of Campania “Luigi Vanvitelli”, Naples, Italy; ^7^ Department of Experimental, Clinical and Biomedical Sciences “Mario Serio”, University of Florence, Florence, Italy; ^8^ Department of Maternal and Child Health, Careggi University Hospital, Florence, Italy; ^9^ Department of Obstetrics, Gynecology, Gynecologic Oncology and Senology, Bethesda Hospital Duisburg, Duisburg, Germany; ^10^ Faculty of Mathematics and Computer Science, University of Bremen, Bremen, Germany

**Keywords:** adenomyosis, hormone treatment, dienogest, levonorgestrel intrauterine device, combined oral contraceptives, chronic pelvic pain, abnormal uterine bleeding, quality of life

## Abstract

**Background:**

To date, there are no clear guidelines available on the treatment of adenomyosis-associated pelvic pain (AAPP); however, numerous hormonal treatments (HTs) are currently being used off-label. We conducted a systematic review and network metanalysis with the aim of assessing the efficacy and safety of HTs to reduce AAPP and ranking the available options.

**Methods:**

MEDLINE, LILACS, EMBASE, Scielo.br, PROSPERO, Cochrane Library, conference proceedings, and international registries were searched with no time, region, or language restrictions. Randomized controlled trials that analyzed AAPP in women undergoing HTs were deemed suitable.

**Results:**

Six studies (563 women affected by adenomyosis) were included. At 3 months, women who received a placebo or a levonorgestrel-based intrauterine system (LNG-IUS) experienced more AAPP than those who received dienogest [mean difference of visual analog scale (VAS) pain scores (MD) 4.10 (95% CI 0.49 to 7.71); high evidence; MD 3.05 (95% CI 0.45 to 5.65); high evidence]. At 6 months, women who received dienogest experienced significantly less AAPP compared to those who received combined oral contraceptives [MD -2.85 (95% CI -5.30 to -0.39); moderate evidence], while the prevalence of AAPP was higher among those who received a LNG-IUS than those who received dienogest [MD 1.79 (95% CI 0.06 to 3.53); low evidence].

**Conclusion:**

Dienogest seems to be the most effective HT for AAPP. However, although rare, it is also related to more adverse effects compared to other HTs.

**Systematic Review Registration:**

https://www.crd.york.ac.uk/prospero/, identifier CRD42024535472.

## 1 Introduction

Adenomyosis is a benign, estrogen-dependent inflammatory pathology of the myometrium frequently seen in the female population of childbearing age (1). Its prevalence is variable and is estimated to vary from 5% to 70% (2), depending on the target population and the diagnostic method, either transvaginal ultrasound (TVUS), magnetic resonance imaging (MRI), or histology (3). Its genesis is not yet adequately explained, although it is speculated that the onset may be due to the disruption of the normal junction between the basal endometrium and myometrium (4, 5). Clinical issues related to adenomyosis include impaired quality of life (QoL) due to severe chronic pelvic pain (CPP), abnormal uterine bleeding (AUB), heavy menstrual bleeding (HMB), infertility, recurrent pregnancy loss, and demanding appropriate treatment (6).

To date, there are no clear guidelines available on the treatment of adenomyosis. However, similar to endometriosis, numerous hormone treatments are currently being used off-label to control the symptomatology (7) and to avoid the excessive recourse to hysterectomy that has been reported in the recent past (8). The most used hormone treatments include progestins, combined oral contraceptives (COCs), hormone-releasing intrauterine systems (IUSs), and gonadotropin-releasing hormone (GnRH) agonists and antagonists (9). However, the superiority of one treatment over another in terms of efficacy and safety has not yet been clarified.

The present systematic review and network meta-analysis aims to explore the superiority of one hormone treatment over others in terms of efficacy in controlling adenomyosis-related pain symptoms and safety in its use by analyzing the currently available randomized controlled trials (RCTs) conducted for this purpose.

## 2 Materials and methods

This network meta-analysis was performed according to the principles included in the Cochrane Handbook for Systematic Reviews of Interventions (10) and the methodological specifications by Mbuagbaw et al. (11). It adhered to the Preferred Reporting Items for Systematic Reviews and Meta-Analyses (PRISMA) extension statement for network meta-analyses (PRISMA-NMA) (12). Due to its design (systematic review with a network meta-analysis), the study was deemed exempt from ethical approval. The study protocol was registered in the International Prospective Register of Systematic Reviews (PROSPERO) database (CRD42024535472) on 26/04/2024.

### 2.1 Eligibility criteria, information sources, search strategy

MEDLINE (available via PubMed), LILACS, EMBASE, Scielo.br, and PROSPERO are among the electronic databases that were searched using the following keywords and Medical Subject Heading (MeSH) phrases: “adenomyosis” (MeSH Unique ID: D062788) AND “hormone therapy” OR “hormone treatment” without any date restriction. The search string was modified according to each database’s format (**Supplementary Table S1**). A filter was applied to the search results to display only RCTs. Searches were also conducted on CINAHL, PsycINFO, and AMED to reduce publication bias by finding more relevant papers. The World Health Organization’s International Clinical Trials Registry Platform (ICTRP), Cochrane Central Register of Controlled Research, and Clinicaltrials.gov were also examined to locate more randomized controlled trials. Furthermore, a search of the grey literature (NTIS and PsycEXTRA) was conducted to find conference abstracts at the national and international levels. References of the included studies and related reviews were also searched to discover other articles that had been overlooked in the initial search. There were no limitations according to language or location. The search did not include editorials, letters to the editor, comments, or second opinions.

### 2.2 Study selection

The inclusion criteria considered any randomized controlled trial that enrolled women diagnosed with adenomyosis undergoing hormone treatment. Adenomyosis was diagnosed in all included studies either by ultrasound according to the terms and definition of the Morphological Uterus Sonographic Assessment (MUSA) group (13) or by MRI. Studies were included if they evaluated treatment with any hormone-based therapy. Intervention groups had to be compared among them or to a placebo/no treatment arm. The exclusion criteria were quasi-randomized trials, trials without randomization, and studies that included non-hormone interventions (e.g., surgery, non-hormone medical treatments including selective progesterone or estrogen receptor modulators, and dietary supplements).

### 2.3 Outcomes

The co-primary outcomes of this network meta-analysis were the adenomyosis-associated pelvic pain (AAPP) at 3 and 6 months, defined for the included studies as CPP related to the disease and assessed through a visual analog scale (VAS). The VAS is a horizontal line with a length of 10 cm anchored at both ends, with 0 denoting no pain and 10 reflecting the highest pain level. In the case of studies reporting a VAS ranging from 0 to 100, such reports were re-scaled to a 0 to 10 VAS scale. The secondary outcomes were the assessment of the uterine volume change at 6 months, defined as the global volume of the uterus and assessed using the standardized formula for an ovoid object (length × width × depth × 0.52), and the evaluation of common hormone-related adverse effects, including irregular uterine bleeding, breast tenderness, and hot flashes.

### 2.4 Data extraction

The abstraction forms were explicitly created for this network meta-analysis. The following important facts were noted: patient descriptions, study duration, setting, adenomyosis characteristics, treatment types, outcomes assessed, mean treatment and follow-up time, results, and quality elements. Two authors (AE and GR) examined and categorized each abstract independently. In order to come to a consensus on potential relevance, the same two authors thoroughly analyzed the texts of the selected studies. They independently gathered pertinent information about the research characteristics and the noteworthy findings. After discussing each contradiction with the other three authors (A.S.L., V.C., and S.V.), the reviewers reached a consensus. Unpublished data were obtained by contacting the authors of the original studies directly when the study procedures indicated that additional outcome data were collected.

### 2.5 Assessment of risk of bias

The Cochrane Handbook for systematic reviews of interventions’ criteria by means of the RoB 2 Tool (10) were applied to assess the risk of bias in every included study. The critical investigation of each included trial concentrated on the following five domains: bias resulting from the random process, bias that deviates from the prescribed intervention, bias associated with incomplete outcome data, bias associated with outcome assessment, and bias associated with selective results reporting. It was ascertained whether the writers’ evaluations carried a “low risk,” “high risk,” or “unclear risk” of bias. The risk of bias assessment was rated independently by three authors (V.A., A.D., and L.C.). The disagreement was resolved after speaking with other reviewers (P.D.F., J.H., A.N., and H.K.). The certainty of evidence was assessed using the Confidence in Network Meta-Analysis framework (CINeMA) criteria (14). Considering an overall number of included studies fewer than 10, publication bias was not assessable either with a funnel plot analysis or with an Egger’s test.

### 2.6 Data synthesis

The summary measures were presented as mean differences (MDs) for continuous variables and odds ratios (ORs) for categorical variables, with 95% confidence intervals (CIs), using the Der Simonian and Laird random effects model. A Higgins I2 score was used to identify potential heterogeneity where 25%, 50%, and 75% were used as the thresholds for low, intermediate, and high heterogeneity, respectively.

All data analysis and graphical representations were performed using STATA version 14.1 (StataCorp, College Station, TX). For every outcome, the network assumption of overall consistency was statistically tested using the command <network meta consistency>. The local test on loop inconsistencies was then conducted using the command <network meta inconsistency> and the Separating Indirect from Direct Evidence (SIDE)-splitting technique using the command <network sidesplit all>. When no discrepancy was found in either the global or local tests, the study’s direct and indirect comparisons would undoubtedly yield meaningful results. For each outcome under investigation, a ranking plot [Surface Under the Cumulative Ranking curve Area (SUCRA)] and a prediction interval plot were created to assess the effectiveness of the various hormone regimens and rank the therapies to establish which is superior. A p-value (p) <.05 was considered statistically significant.

## 3 Results

### 3.1 Study selection

**Figure 1:**
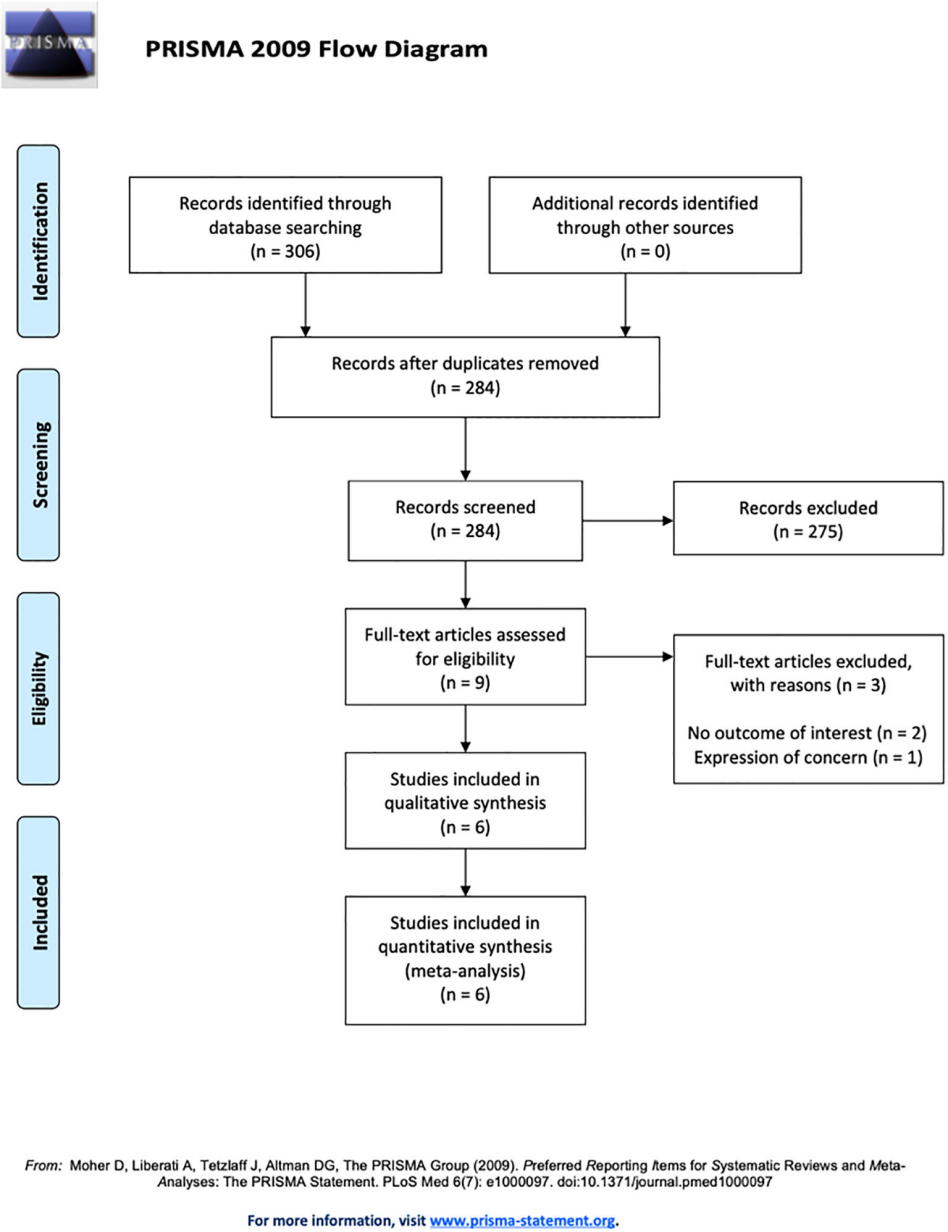
PRISMA flowchart of the included studies in the systematic review and network meta-analysis.

In total, 306 studies were initially identified using database searches. Of these, 22 were removed as duplicates. After title and abstract screening, 275 papers were subsequently removed (**Figure 1**). Nine studies underwent full-text assessment; two were excluded for not reporting a treatment of interest (**Figure 1**). Another study was excluded before the final analysis due to an expression of concern related to the article’s content. Six studies (15–20), including 563 women affected by adenomyosis randomized to treatment or control groups, were included in the systematic review and network meta-analysis (**Figure 1**).

### 3.2 Study characteristics

The main characteristics of the included studies are summarized in **Table 1**. The inclusion and exclusion criteria and the methodology adopted by the included studies are summarized in **Supplementary Table S2**. All the studies were randomized controlled trials (15–20). Two studies came from Egypt (15, 17), two from Japan (16, 18), one from China (19), and one from India (20).

**Table 1:** Characteristics of studies included in the systematic review and network meta-analysis.

Author	Year	Type	Main outcome	Country	Patient (n)	Months of therapy	Results	Trial registration
Shabaan et al. (16)	2015	RCT	• **Primary outcome**: improvement of pelvic pain (dysmenorrhea and or chronic pelvic pain) as measured using a VAS. • **Secondary outcome**: menstrual blood loss as measured using the menstrual diary.	Egypt	57 • COC group: 28. • LNG-IUS group: 29.	1	• Both treatments significantly reduced pain after 6 months of use; however, the reduction was greater in the LNG-IUS group (from 6.23±0.67 to 1.68±1.25) compared with the COCs group (from 6.55±0.68 to 3.90±0.54). • Both treatment arms significantly decreased the number of bleeding days, uterine volume and Doppler blood flow in the uterus from before to after treatment. These effects were more significant in the LNG-IUS arm compared with the COC arm.	NCT01601366
Osuga et al. (17)	2017	RCT	• **Primary outcome**: the change in the pain score from baseline to after treatment. • **Secondary outcome**: the change in the pain score during treatment and change in the VAS and uterine size from baseline to after treatment. • **Tertiary outcome**: adverse effects (AEs) and adverse drug reactions (ADRs).	Japan	67 • DNG group: 34. • Placebo group: 33.	4	• Decreases from baseline in the pain score and the visual analogue scale at the end of treatment were significantly more in the DNG group than in the placebo group (P< .001). • During the treatment period, almost all of the patients treated with DNG experienced irregular uterine bleeding and one patient had mild anemia. • No severe cases of anemia were observed.	JapicCTI-142642(en)
Hassanin et al. (18)	2020	RCT	• **Primary outcome**: The level of adenomyosis-associated pain from before to 6 months after treatment measured by VAS. • **Secondary outcome**: The menstrual pattern, uterine and ovarian volumes, uterine artery Doppler indices, reported adverse effects, patient satisfaction, and hemoglobin level.	Egypt	97 • DNG group: 49. • COC group: 48.	6	• The VAS score of pain was significantly decreased in both groups; however, the decreased rate was more pronounced in the DNG group (3.21±1.18) in com- parison with the COCs group (4.92±1.22). • Bleeding pattern was improved greatly. • Uterine volume and uterine artery blood flow decreased significantly in the DNG group. • Higher rate of side effects in the DNG group.	NCT03890042
Ota et al. (19)	2021	RCT	• **Primary outcome**: The change in pain score from baseline to after treatment. • **Secondary outcome**: assessment of bleeding days, uterine volume and bone density before and after treatment.	Japan	157 • DNG group: 81 (Focal: 23, Diffuse: 28, Extrinsic: 30). • LNG-IUS group: 76 (Focal: 21, Diffuse:27, Extrinsic: 28).	72	• LNG-IUS and DNG comparably reduced pain scores in patients with adenomyosis. • Pain control, DNG offered greater efficacy than LNG-IUS in 3 months of treatment. • In all types of adenomyosis, the days of bleeding after 12 months with DNG were significantly decreased compared to those with LNG-IUS. • The decrease of whole uterine body was transient in any subtypes. • A comparable decrease in BMD due to age-related changes in both groups was observed.	None
Guo et al. (20)	2023	RCT	• **Primary outcome**: to investigate the efficacy and safety of LNG-IUS versus DNG in symptomatic female subjects with symptomatic uterine adenomyosis.	China	117 • DNG group: 69. • LNG-IUS: 48.	36	• The VAS pain score was significantly decreased in both groups after 3 months of treatment. • At the 3 months mark, patients receiving DNG reported significantly lower VAS scores compared with those treated with LNG-IUS(P <0.05). • Compared with LNG-IUS, DNG effectively controlled uterine volume growth after 12 months of treatment but neither significantly reduced uterine volume. • During the treatment period, endometrial thickness in both groups was maintained at 0.4 to 0.7cm.	None
Choudhury et al. (21)	2024	RCT	• **Primary outcome**: the change in the level of adenomyosis-associated pelvic pain(dysmenorrhea or CPP) from before treatment to 12weeks after treatment, measured by VAS. • **Secondary outcome**: change in menstrual pattern, change in quality of life, and reported adverse drug reactions.	India	68 • DNG group: 34. • LNG-IUS group: 34	3	• Both groups showed significantly reduced pelvic pain (VAS scores), but no significant difference was found between the groups. • LNG-IUS resulted in a significantly greater reduction in HMB. • DNG showed better improvement in overall QoL. • Adverse effects were similar in both groups, with hot flushes reported in the DNG group.	CTRI/2020/05/025186

RCT: randomized controlled trial; VAS: Visual analogue scale; COC: combined oral contraceptive; LNG-IUS: levonorgestrel Intrauterine system; DNG: dienogest; HMB: heavy menstrual bleeding; QoL: quality of life; BMD: bones mineral density.

COCs, dienogest (DNG), a levonorgestrel-based intrauterine system (LNG-IUS), and placebo were used in the included RCTs. Three studies randomized patients between a DNG arm and an LNG-IUS arm (18–20), one study randomized patients between a DNG arm and a COC arm (17), one study randomized patients between a DNG arm and a placebo arm (16), and another one randomized patients between a COC arm and an LNG-IUS arm (15). A total of 267 patients received DNG, 76 patients received a COC, 187 patients received an LNG-IUS, and 33 patients received a placebo.

### 3.3 Risk of bias of included studies

The risk of bias assessment in each experiment is shown in **Supplementary Figure S1A**; a percentage-based assessment of the methodology’s quality for all trials is illustrated in **Supplementary Figure S1B**. There was a low chance of bias in most of the research that made up the analysis. However, because staff and participant blinding controls were unclear in four of the six studies, the control-related scores indicated moderate risk. In addition, two out of six studies (19, 20) did not report information regarding the randomization process (**Supplementary Figures S1A, B**). Four of the included studies were documented in valid prospective registries before participant participation. In two studies (18, 19), no information regarding the trial registration was available (**Table 1**). For most of the included comparisons, according to CINeMA criteria, high-certainty evidence was retrieved. However, low to very low evidence was found for certain evaluations concerning minor outcomes. A detailed comparison-by-comparison evaluation is presented in Appendix 1. We were unable to assess the publication bias as less than 10 studies were included in the final analysis.

### 3.4 Synthesis of results

#### 3.4.1 AAPP after 3 months of treatment

**Figure 2:**
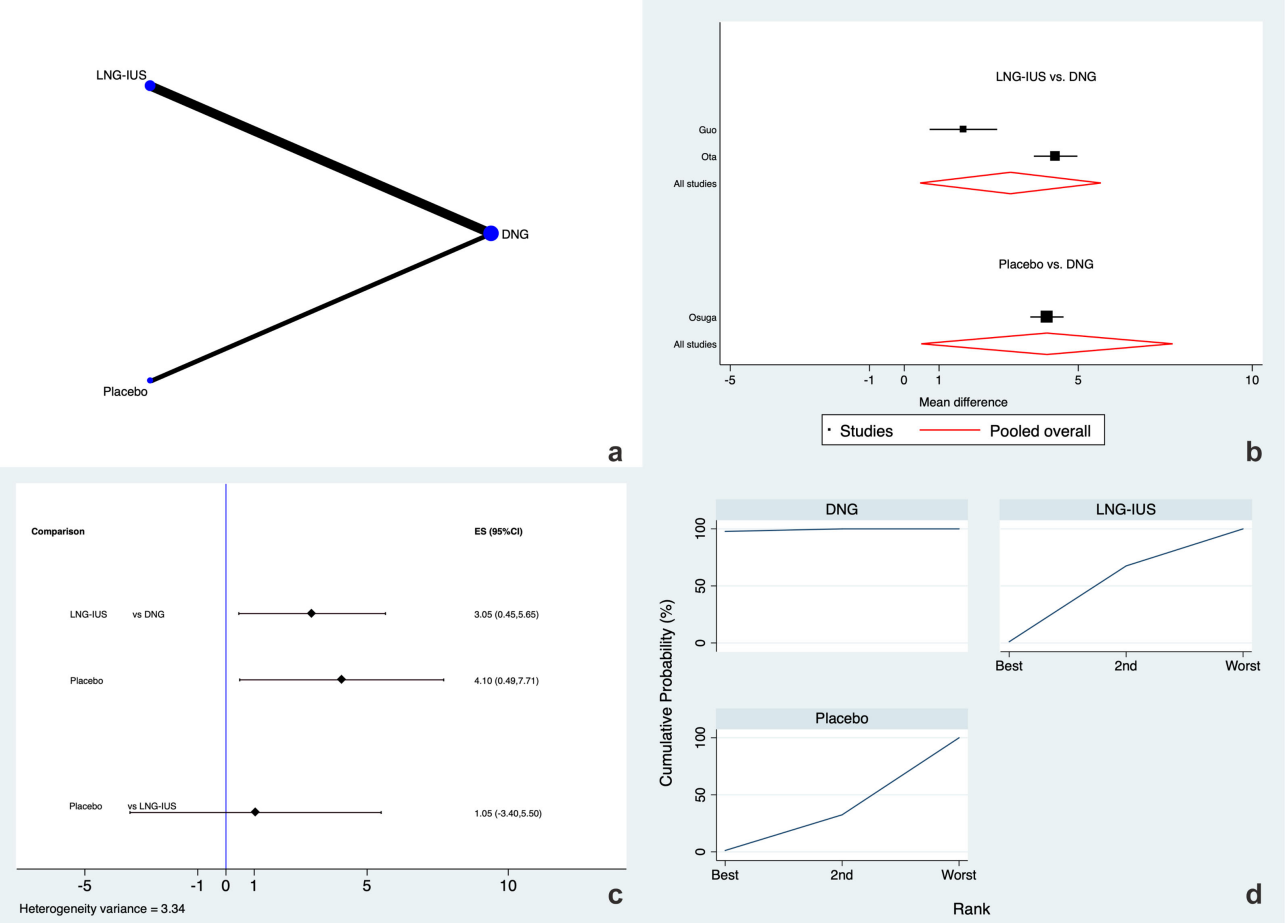
Adenomyosis-associated pelvic pain at 3 months **(a)** Network of comparisons of the interventions analyzed in the included studies. **(b)** Forest plot of the outcome. **(c)** Prediction interval plot. **(d)** Ranking plot according to SUCRA analysis.

AAPP after 3 months of treatment was analyzed by three studies (16, 18, 19). DNG, LNG-IUS, and placebo were directly and indirectly evaluated. The most accurate direct comparisons and the frequency of examined therapies are displayed in **Figure 2a**. There were no sources of inconsistency and therefore the SIDE analysis for local inconsistency was not performable. **Figures 2b, c** show the forest plot and predictive interval plot, respectively, which showed the effect of various techniques on the decrease in AAPP. According to these studies, women who received a placebo or an LNG-IUS experienced significantly more AAPP than women who received DNG, reporting a mean difference of approximately four and three points when placebo or LNG-IUS were used instead of DNG, respectively [MD 4.10 (95% CI 0.49 to 7.71), high evidence; and MD 3.05 (95% CI 0.45 to 5.65), high evidence respectively]. According to the SUCRA ranking (**Figure 2d**), DNG had the highest possibility of being the treatment of choice for reducing AAPP at 3 months (SUCRA=97.8%) relative to placebo (SUCRA=1.2%) and LNG-IUS (SUCRA=1.0%).

#### 3.4.2 AAPP after 6 months of treatment

**Figure 3:**
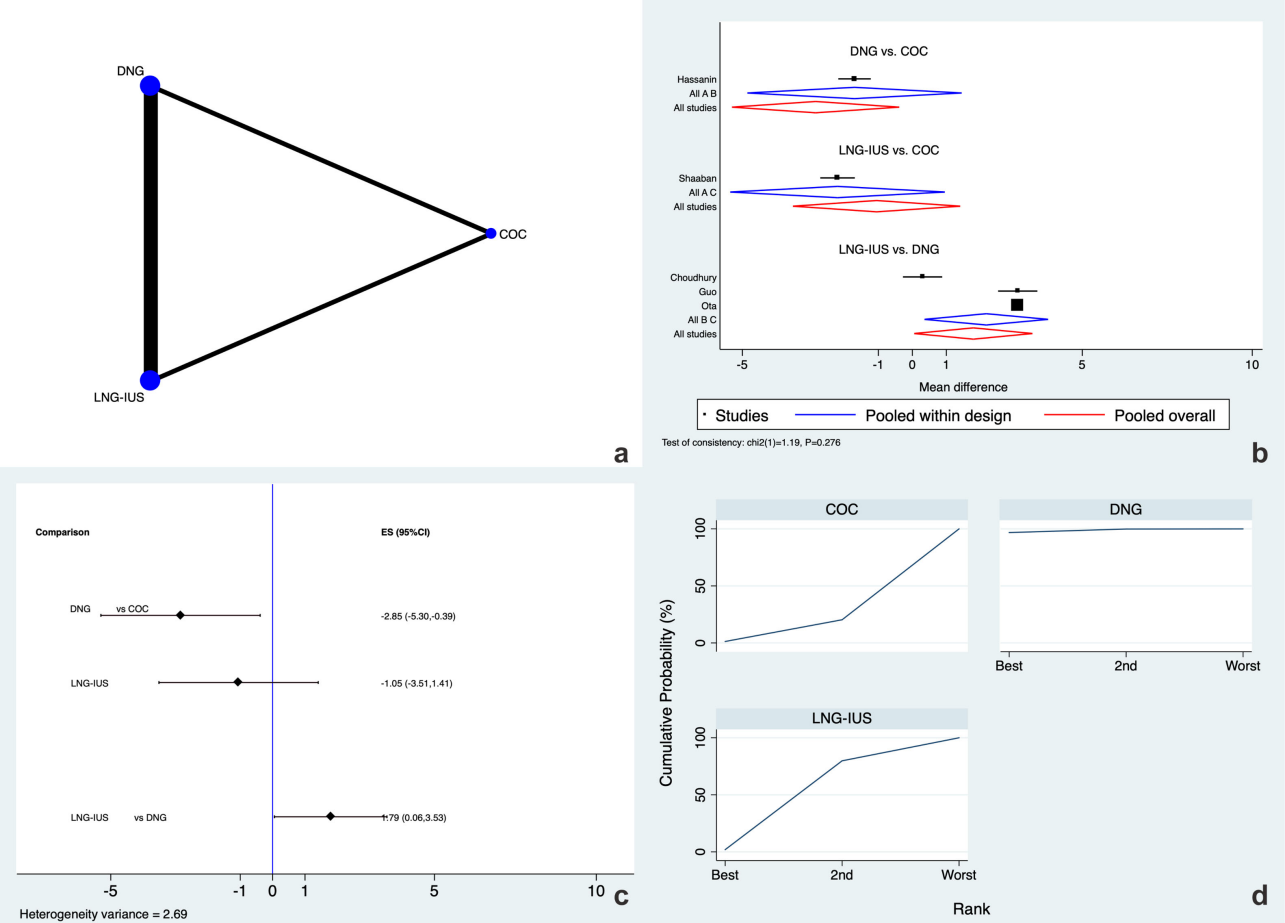
Adenomyosis-associated pelvic pain at 6 months. **(a)** Network of comparisons of the interventions analyzed in the included studies. **(b)** Forest plot of the outcome. **(c)** Prediction interval plot. **(d)** Ranking plot according to SUCRA analysis.

AAPP after 6 months of therapy was evaluated by five RCTs (15, 17–20), which analyzed the influence of DNG, COC, and LNG-IUS (**Figure 3a**). Global inconsistency for this outcome was not retrievable (p=0.276). The closed loops tested for this outcome showed no local inconsistency (**Supplementary Table S3**). The analyses of the network forest plot and interval plot (**Figures 3b, c**) showed that women who received DNG experienced significantly less AAPP than women using COCs, reporting almost three points less in the VAS score [MD -2.85 (95% CI -5.30 to -0.39), moderate evidence]. Meanwhile, more pain, almost two points in the VAS score, was reported by women who received an LNG-IUS compared to DNG usage (MD 1.79 [95% CI 0.06 to 3.53]; low evidence). No difference was noted between COC and LNG-IUS (**Figure 3c**). According to the SUCRA analysis (**Figure 3d**), DNG was ranked as the treatment of choice for reducing AAPP at 6 months (SUCRA=96.8%) against COC (SUCRA=1.3%) and LNG-IUS (SUCRA=1.9%).

#### 3.4.3 Uterine volume

**Figure 4:**
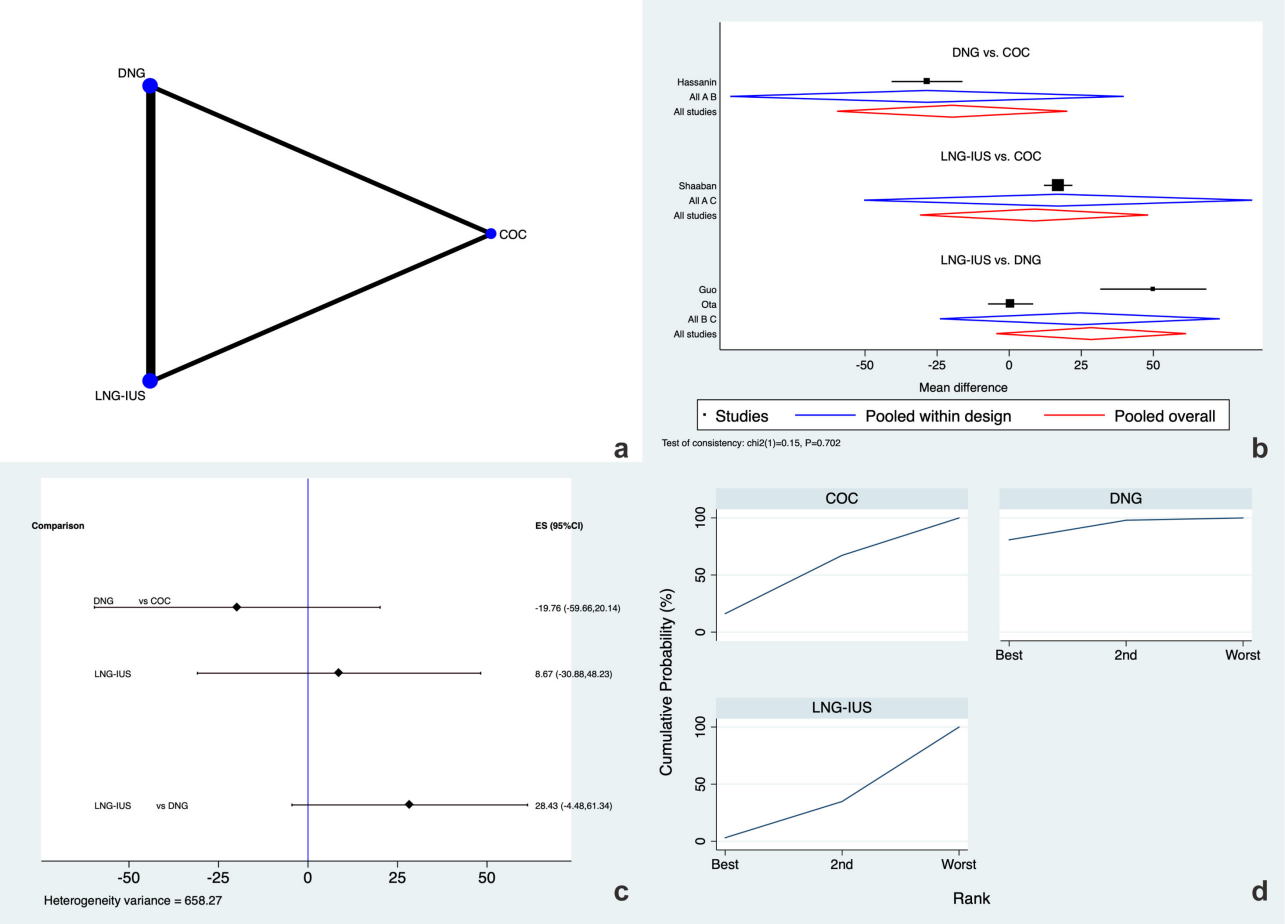
Uterine volume at 6 months. **(a)** Network of comparisons of the interventions analyzed in the included studies. **(b)** Forest plot of the outcome. **(c)** Prediction interval plot. **(d)** Ranking plot according to SUCRA analysis.

Uterine volume after 6 months of treatment was analyzed in four RCTs (15, 17–19). DNG, COC, and LNG-IUS were directly and indirectly compared (**Figure 4a**). No inconsistency was retrievable for this outcome (p=.702), and there were no local inconsistencies in the examined closed loops (**Supplementary Table S3**). There were no differences in MD between the three hormone treatments according to the evaluation of the network forest plot and prediction interval plot (**Figures 4b, c**). The SUCRA analysis revealed that DNG (SUCRA=80.8%), rather than COC (SUCRA=16.2%) and LNG-IUS (SUCRA=3.0%), had higher chances of being ranked first for this outcome (**Figure 4d**).

#### 3.4.4 Adverse effects

**Supplementary Table S4** summarizes the adverse effects of the evaluated treatments. For all the comparisons, no sources of inconsistency were retrievable (p=.999). Patients using DNG were more significantly exposed to irregular uterine bleeding and hot flashes compared to women using COCs. Similarly, women in the placebo arm were significantly less prone to them relative to women treated with DNG or an LNG-IUS. Additionally, irregular bleeding was more present among the women using LNG-IUSs compared to COCs (**Table 2**). According to SUCRA analysis, COC usage was more likely to be the treatment of choice for avoiding irregular uterine bleeding, hot flashes, and breast tenderness (SUCRA= 50.3%, 69.8%, and 76.7% respectively). Conversely, DNG had the lowest chances of being first according to adverse effect presence (**Supplementary Table S4**).

**Table 2:** Effect sizes related to interventions by means of direct and indirect comparisons.

	DNG	LNG-IUS	COC	
**DNG**			**52.13 (7.29 to 372.66)** **28.97 (1.43 to 587.18)** 2.15 (0.60 to 7.67)	Irregular bleeding Hot flashes Breast tenderness
**LNG-IUS**	0.92 (0.31 to 2.73) 0.32 (0.01 to 8.31) 0.96 (0.33 to 2.77)		**47.80 (5.04 to 452.95)** 9.38 (0.11 to 825.69) 2.05 (0.39 to 10.79)	Irregular bleeding Hot flashes Breast tenderness
**Placebo**	**0.02 (0.00 to 0.23)** 0.19 (0.01 to 4.26) NA	**0.02 (0.00 to 0.31)** 0.60 (0.01 to 52.04) NA	1.03 (0.04 to 23.53) 5.62 (0.07 to 447.25) NA	Irregular bleeding Hot flashes Breast tenderness

Results are reported as OR and 95% confidence intervals.

COC: combined oral contraceptive; LNG-IUS: levonorgestrel Intrauterine system; DNG: dienogest; NA: not available.

Bold values: statistically significant.

## 4 Discussion

The results of this systematic review and network meta-analysis suggest that hormone therapies are effective in treating AAPP. Among these, DNG has the highest chance of being considered the treatment of choice to reduce AAPP at 3 and 6 months. A mean of four and three points less on a 10-point VAS scale was reported at 3 months using DNG compared to placebo and LNG-IUS and a mean of almost 3 and 2 out of 10 at 6 months. COCs, however, according to the results of our study, have the lowest chance of presenting adverse effects, and thus they would seem to have the highest chance of being considered the treatment of choice in terms of tolerability by patients, increasing treatment adherence and the physician-patient alliance. Recent reviews have shown that medical treatment options for adenomyosis are as varied as ever, but all of these are considered off-label (7, 21).

DNG and LNG-IUS have been classically referred to as the drugs of first choice for this condition, implying, however, that the choice between one drug and the other may depend solely on the patient’s tolerance or the physician’s personal choice rather than the superiority of one over the other (22). Though DNG would seem to be the best treatment at 3 and 6 months and the one that should be considered the first treatment option, it is interesting to analyze the data on LNG-IUSs and COCs. Indeed, comparative analysis of the latter shows that their efficacy in the treatment of AAPP at 6 months would appear to be comparable, thus increasing the potential for the use of COCs; consequently, COCs should no longer be viewed as a less effective alternative to an LNG-IUS, but instead as its equal. Although these results are consistent with those reported by Hassanin et al. (17), they would, however, seem discordant when compared only to the randomized controlled trial conducted by Shaaban et al. (15), who, despite reporting an actual efficacy of COCs, also emphasized its lesser efficacy compared to LNG-IUSs. This could be related to progressive dysmenorrhea being the main characteristic for the definition of AAPP in Hassanin et al.’s trial (17).

Uterine volume is also an issue that needs to be considered and managed in patients with adenomyosis (23). In addition to further worsening AAPP, the latter generates major pelvic venous stasis problems (24) and especially issues pertaining to the reproductive sphere (23). Despite the promising efficacy of DNG in the treatment of adenomyosis, the studies in the literature available today appear to be cohesive in stating that it is often poorly tolerated by patients (16–20, 25, 26), and therefore, LNG-IUSs (18–20) and especially COCs (17) can be used as alternatives with reduced incidence of side effects. An LNG-IUS, due to reduced serum hormone levels and locally high concentrations of LNG in the endometrium and adjacent tissues, according to many authors, is better tolerated than DNG (27).

This network meta-analysis has certain limitations that should be noted. There were a few studies, with a single-center design, that satisfied the inclusion criteria. Though independent participants were included, there is a greater risk for selection, performance, detection, and reporting biases which limit the generalizability of the studies’ results. It should be noted that the pharmaceutical effects of the hormonal agents and the adenomyosis characteristics may differ based on the genetic and epigenetic profiles of the participants. For specific outcomes, most of the trials were conducted in single centers. Though independent participants were included, there is a greater risk for selection, performance, detection, and reporting biases relative to multiple-center studies. Although the differences in study duration unpredictability increased heterogeneity, the longer length of follow-up should theoretically reduce AAPP to improve therapy compliance, resulting in different results, which is an advisable limitation of our study.

This network meta-analysis has certain limitations that should be noted. There were a few studies that satisfied the inclusion criteria, and the sample sizes needed to be increased. The low sample size of included studies may lead to selection bias and limit the generalizability of the studies´ results. It should be noted that the pharmaceutical effects of the hormonal agents and the adenomyosis characteristics may differ based on the genetic and epigenetic profiles of the participants. Furthermore, the small sample sizes may have led to investigating the hormonal agents’ effects on adenomyosis-associated pain in specific groups of patients. Thus, the low number of available studies may indicate publication bias, since authors tend to publish positive results only. This may lead to an overestimation of the hormonal therapies effect on adenomyosis.

Although the differences in study duration unpredictability increased heterogeneity, the longer length of follow-up should theoretically reduce AAPP to improve therapy compliance, resulting in different results. Nonetheless, the short follow-up period may hide a washout effect and a decreased effectiveness of the hormonal therapies upon the discontinuation of the treatment. Another potential drawback is developing tolerance against the hormonal agents, which may lead to symptoms recurrence over the long term. This is also an advisable limitation of our study.

Furthermore, we could not assess the role of endometriosis among the potential confounders since only Hassanin et al. (17) excluded women with concomitant endometriosis. One of the study’s several advantages is its capacity to extrapolate its conclusions to various geographical areas. However, the primary strength of our study lies in the caliber of the literature we consulted, as the quantitative analysis only comprised randomized trials with a low overall risk of bias. Different from the systematic review from Rathinam et al. (23), there were no observational studies, open trials, quasi-randomized trials, or single-arm studies included, strengthening the robustness of the evidence. Future research should include clinical trials with long follow-up periods and large sample sizes. It is advisable to directly compare the hormonal therapies to each other to decide which is superior in terms of efficacy, tolerability, and adverse effects profiles. In addition, it is important to precisely estimate the effect size of each hormonal therapy and estimate its cost-effectiveness. Finally, the efficacy of letrozole, GnRH agonists, and the new generation of GnRH antagonists along with their adverse effects should be investigated with robust clinical trials.

## 5 Conclusions

Various treatments can be used for AAPP relief, and even if DNG seems the most effective, it is also related to more unpleasant adverse effects. However, the overall adverse effect rate was low in each included trial, emphasizing the need for additional evidence on such an issue. Our study lays the basis for the thoughtful selection of a therapeutic agent according to its efficacy and tolerability by the patient. Nevertheless, since the number of studies included was small, therefore, this conclusion must be referenced cautiously. Further high-quality, adequately designed randomized controlled trials are needed to evaluate the impact of hormone therapies on patient-centered outcomes.

## Data Availability

The original contributions presented in the study are included in the article/**Supplementary Material.** Further inquiries can be directed to the corresponding author.
