# Supplementary material for: Efficacy and safety of hormone therapies for treating adenomyosis-associated pelvic pain: a systematic review and network meta-analysis of randomized controlled trials
Source: Front Endocrinol (Lausanne). 2025 Mar 17;16:1571727. doi: 10.3389/fendo.2025.1571727 (PMC11955467; doi:10.3389/fendo.2025.1571727)
Supplement: Supplementary file 1 [file DataSheet1.docx]

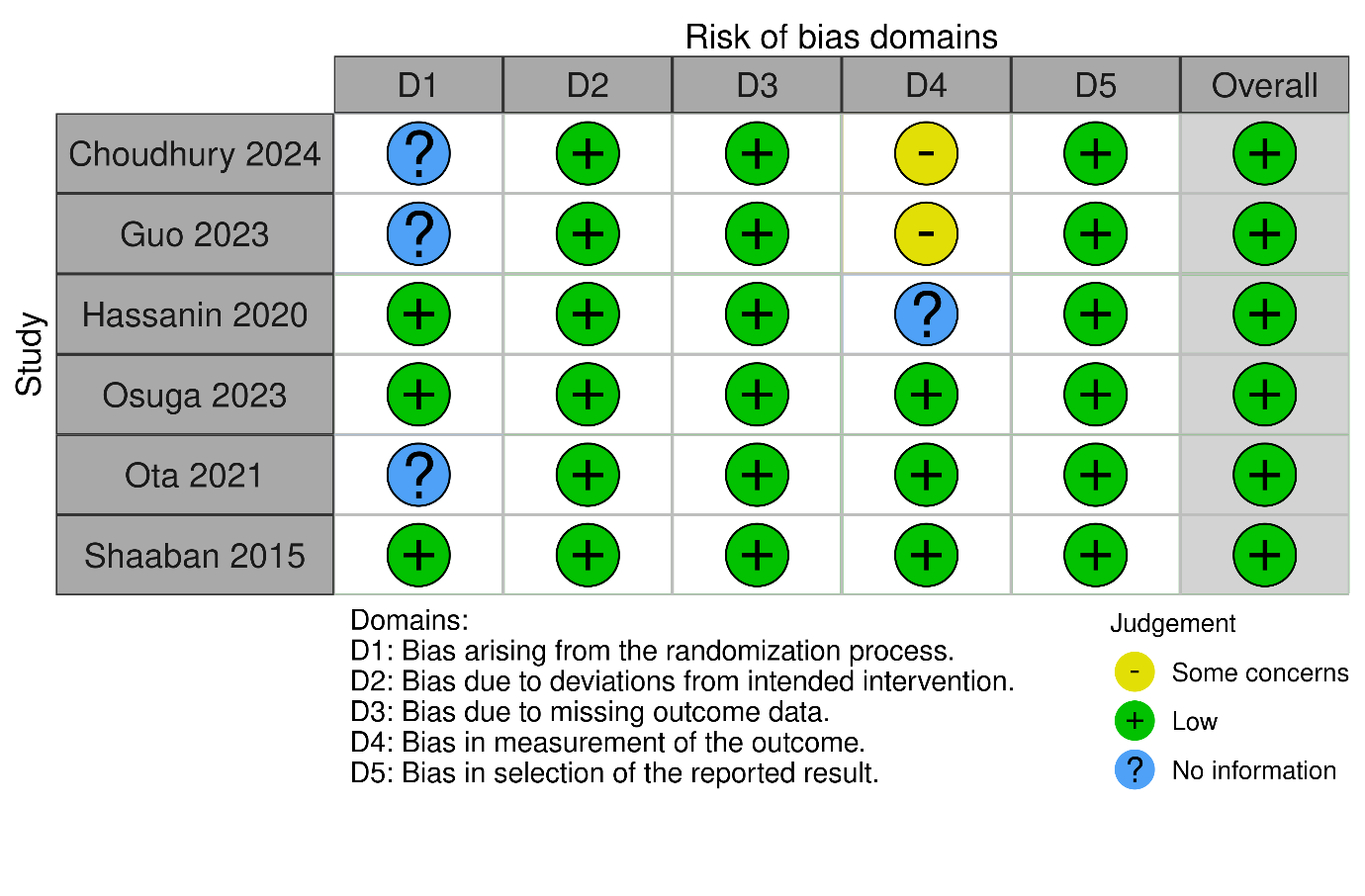


**Figure S1a.** Assessment of risk of bias.

Summary of risk of bias for every trial; plus sign: low risk of bias; minus sign: some concerns; question mark: no information.
