# Supplementary material for: Efficacy and safety of hormone therapies for treating adenomyosis-associated pelvic pain: a systematic review and network meta-analysis of randomized controlled trials
Source: Front Endocrinol (Lausanne). 2025 Mar 17;16:1571727. doi: 10.3389/fendo.2025.1571727 (PMC11955467; doi:10.3389/fendo.2025.1571727)
Supplement: Supplementary file 2 [file DataSheet2.docx]

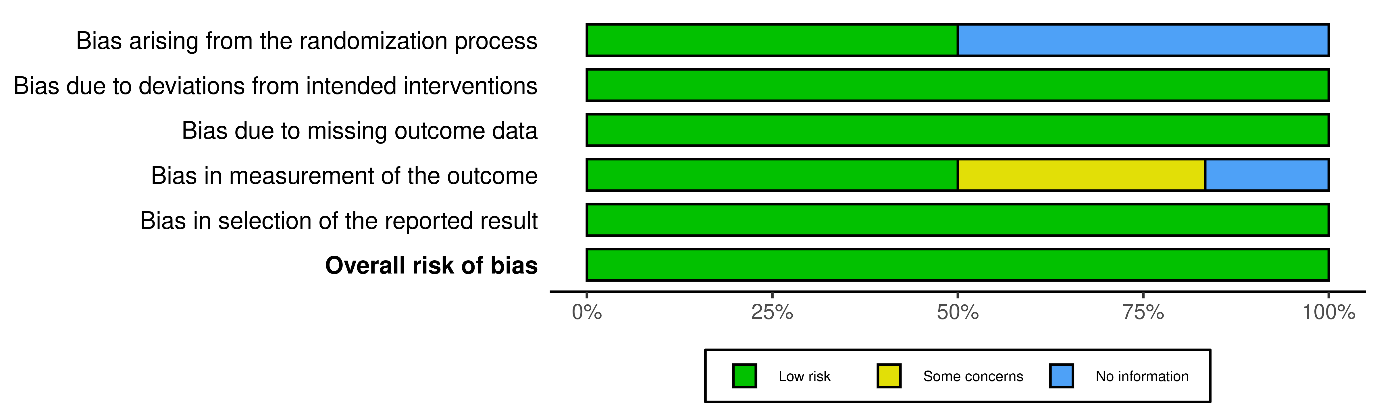


**Figure S1b.** Assessment of risk of bias.

Risk of bias graph about each risk of bias item presented as percentages across all included studies.
