# Supplementary material for: Efficacy and safety of hormone therapies for treating adenomyosis-associated pelvic pain: a systematic review and network meta-analysis of randomized controlled trials
Source: Front Endocrinol (Lausanne). 2025 Mar 17;16:1571727. doi: 10.3389/fendo.2025.1571727 (PMC11955467; doi:10.3389/fendo.2025.1571727)
Supplement: Supplementary file 3 [file Table1.docx]

| **MEDLINE** (accessed through PUBMED)  ("adenomyosis"[MeSH Terms] OR "adenomyosis"[All Fields] OR "adenomyoses"[All Fields]) AND ((("hormon"[All Fields] OR "hormonal"[All Fields] OR "hormonally"[All Fields] OR "hormonals"[All Fields] OR "hormone s"[All Fields] OR "hormones"[Pharmacological Action] OR "hormones"[MeSH Terms] OR "hormones"[All Fields] OR "hormone"[All Fields] OR "hormons"[All Fields]) AND ("therapeutics"[MeSH Terms] OR "therapeutics"[All Fields] OR "therapies"[All Fields] OR "therapy"[MeSH Subheading] OR "therapy"[All Fields] OR "therapy s"[All Fields] OR "therapys"[All Fields])) OR (("hormon"[All Fields] OR "hormonal"[All Fields] OR "hormonally"[All Fields] OR "hormonals"[All Fields] OR "hormone s"[All Fields] OR "hormones"[Pharmacological Action] OR "hormones"[MeSH Terms] OR "hormones"[All Fields] OR "hormone"[All Fields] OR "hormons"[All Fields]) AND ("therapeutics"[MeSH Terms] OR "therapeutics"[All Fields] OR "treatments"[All Fields] OR "therapy"[MeSH Subheading] OR "therapy"[All Fields] OR "treatment"[All Fields] OR "treatment s"[All Fields]))) AND (randomizedcontrolledtrial[Filter]) |
| --- |
| **EMBASE**:  ('adenomyosis'/exp OR adenomyosis) AND ('hormone treatment'/exp OR 'hormone treatment' OR (('hormone'/exp OR hormone) AND ('treatment'/exp OR treatment)) OR 'hormone therapy'/exp OR 'hormone therapy' OR (('hormone'/exp OR hormone) AND ('therapy'/exp OR therapy)))AND ('clinical trial'/de OR 'controlled clinical trial'/de OR 'randomized controlled trial'/de OR 'randomized controlled trial topic'/de) |
| **COCHRANE at CENTRAL**  (adenomyosis) AND ((hormone therapy) OR (hormone treatment)):ti,ab,kw AND ("randomized controlled trial"):pt |
| **CINAHL / PsycINFO / AMED / PsycExtra (accessed through EBSCO – IDEM for Italian Universities)**  (adenomyosis) AND ((hormone therapy) OR (hormone treatment)) Prove controllate randomizzate AND Cerca anche nel testo completo degli articoli; Applica argomenti equivalenti |
| **LILACS**  (adenomyosis) AND ((hormone therapy) OR (hormone treatment)) |
| **Scielo.br**  (adenomyosis) AND ((hormone therapy) OR (hormone treatment)) |
| **Clinicaltrials.gov / ICTRP (accessed through CENTRAL)**  (adenomyosis) AND ((hormone therapy) OR (hormone treatment)):ti,ab,kw AND ("randomized controlled trial"):pt |

**Table S1.** Search query for each database investigated.
