# Supplementary material for: Efficacy and safety of hormone therapies for treating adenomyosis-associated pelvic pain: a systematic review and network meta-analysis of randomized controlled trials
Source: Front Endocrinol (Lausanne). 2025 Mar 17;16:1571727. doi: 10.3389/fendo.2025.1571727 (PMC11955467; doi:10.3389/fendo.2025.1571727)
Supplement: Supplementary file 4 [file Table2.docx]

| Author | Inclusion criteria | Exclusion criteria | Methodology |
| --- | --- | --- | --- |
| Shabaan et al. [16] | - Participant's request for contraception for at least 6 months. - Age between 20 and 45 years. - Resident in the nearby vicinity to make the follow-up easy and feasible. - Acceptance of use of either type of management. | - History of ectopic pregnancy, puerperal sepsis, pelvic inflammatory disease. - Evidence of coagulopathy and/or abnormalities of the uterine cavity such as submucous fibroids distorting the cavity. - History of malignancy or histological evidence of endometrial hyperplasia, any adnexal abnormality on ultrasound, undiagnosed vaginal bleeding or any other contraindication to receive COCs. | Clinically registered open, single centered parallel, RCT comparing the effect of the LNG-IUS (Mirena) with that of low-dose COCs in treating adenomyosis related pain with or without uterine bleeding. The participants were recruited from the Outpatient Gynecology Clinic of the Women's Health Hospital. |
| Osuga et al. [17] | - Age 20 years or older. - Regular menstrual cycles of 38 days or less - Adenomyosis diagnosed by imaging analysis (both MRI and transvaginal sonography). - Pain symptoms (lower abdominal pain and/or lumbago) scoring three points or more on the verbal pain rating scale. | - Endometriosis or uterine leiomyoma diagnosed by imaging analysis (both MRI and transvaginal sonography). - Severe anemia (hemoglobin concentrations <8.0 g/dL, mild anemia was otherwise treated until reaching >= 11.0 g/dL)). - Marked uterine enlargement (maximum dimension, >100.0 mm or myometrial thickness, >40.0 mm). | Phase III, randomized, double-blind, multicenter, placebo-controlled study. Patients were randomly assigned to receive DNG (2mg/d, orally) or placebo for 16 weeks. In cases of complicated anemia, patients were treated for anemia before randomization. |
| Hassanin et al. [18] | - Age 20–45 years - No desire for pregnancy - Accepting prevention of pregnancy (either by COCs or mechanical methods (condom or IUD). - Regular menstrual cycles of 21 to 38 days - Progressive dysmenorrhea (scoring > 4 on VAS/ significant pain). - Adenomyosis diagnosis. - No medical treatment for adenomyosis or hormonal contraceptive methods at least 3 months before recruitment. | - Active pregnancy or breast-feeding. - Refusal of medical treatment - Refusal of COCs, condom or IUD as contraception during the study period. - History or evidence of defective coagulation or gynecological malignancy or severe anemia. - History of Endometriosis diagnosis or uterine leiomyoma. - Any contraindications to COCs or DNG. | Randomized clinical trial including women with symptomatic adenomyosis conducted at Assiut Woman's Health Hospital, Egypt. Participants were randomly assigned to the DNG group (DNG 2 mg/die for 6 months) or COCs group (pills containing 30 μg of Ethinyl estradiol and 75 μg Gestodine die for 21 days followed by a 7-day pill-free interval for 6 months). |
| Ota et al. [19] | - First diagnosis of adenomyosis by transvaginal ultrasound and/or MRI. - Intolerable dysmenorrhea or abnormal uterine bleeding. - No history of moderatedose hormonal pills, GnRHa, testosterone derivatives, estrogen antagonists, aromatase inhibitors, DNG or other progestins, or surgical treatment before enrollment. - No complications of uterine fibroids, including submucosal fibroids. - Age <45 years and pre-menopausal status (follicle-stimulating hormone <11 mIU/ml). - Maximum thickness of myometrium in adenomyosis lesions ≤35 mm. - No history of disease or treatments that affect bone density, such as hypophosphatemia or steroid hormone therapy. | - More than 2-cm ovarian endometrioma in diameter. - The thick adenomyosis with width of more than 35 mm. - The invasive endometriotic lesions to the rectum diagnosed by MRI. | Open-labeled, single-center, randomized clinical trial where women with adenomyosis associated pain were randomized to the LNG-IUS group or the DNG group (2mg die for 72 months). Patients were divided into focal, diffuse and extrinsic group based on adenomyosis types with different result analysis. |
| Guo et al. [20] | - Age ≥18 years. - Regular menstrual cycles of 21 to 38 days. - Pain symptoms (progressive dysmenorrhea or chronic pelvic pain). - Adenomyosis with or without ovarian cyst diagnosed by imaging analysis (transvaginal sonography and/or MRI). | - Active thrombotic disease, moderate to severe anemia, cardiovascular disease, liver and kidney dysfunction, and sex hormone–dependent malignant tumors. - Unexplained genital bleeding. - Receipt of hormone therapy within 3 months before the initiation of study treatment. - Pregnant or lactating women. | Participants were randomized to either the LNG-IUS group (n= 48) or the DNG group (n= 79, 2mg die) in an as controlled clinical trial for 36 months. VAS scores, uterine volume, endometrial thickness, serum CA125 level, estradiol, follicle-stimulating hormone, luteinizing hormone, and side eﬀects were assessed to compare the eﬃcacy of LNG-IUS and DNG. |
| Choudhury et al. [21] | - Women of age >20years. - Pelvic pain symptoms. - Diagnosis of adenomyosis by imaging. | - Ovarian endometrioma or uterine fibroids diagnosed by imaging. - Planned hysterectomy or any other modality of treatment. - Desire for pregnancy in the future. - Contraindications to the use of LNG-IUS or DNG. - Baseline hemoglobin level less than 8 g/dL. | Open-labeled, parallel, single-centered, randomized clinical trial where patients with adenomyosis-associated pain with or without abnormal uterine bleeding were randomly allocated to either LNG-IUS group or DNG group (2mg die for 13 weeks). |

Table S2. Inclusion and exclusion criteria and methodology of included papers.

VAS: Visual analogue scale; COC: combined oral contraceptive; LNG-IUS: levonorgestrel Intrauterine system; DNG: dienogest; GnRHa: gonadotropin-releasing hormone MRI: magnetic resonance imaging.
