# Supplementary material for: Efficacy and safety of hormone therapies for treating adenomyosis-associated pelvic pain: a systematic review and network meta-analysis of randomized controlled trials
Source: Front Endocrinol (Lausanne). 2025 Mar 17;16:1571727. doi: 10.3389/fendo.2025.1571727 (PMC11955467; doi:10.3389/fendo.2025.1571727)
Supplement: Supplementary file 5 [file Table3.docx]

| **AAPP at 3 months**  Side Direct Indirect Difference  Coef. Std. Err. Coef. Std. Err. Coef. Std. Err. P>\|z\|  A B* . . . . . . .  A C* . . . . . . .  *No detectable source of inconsistency |
| --- |
| **AAPP at 6 months**  Side Direct Indirect Difference  Coef. Std. Err. Coef. Std. Err. Coef. Std. Err. P>\|z\|  A B -1.7 1.607982 -4.375927 1.858752 2.675927 2.457756 0.276  A C -2.2 1.61041 .4765993 1.856645 -2.676599 2.457753 0.276  B C 2.176485 .9284736 -.4999996 2.275795 2.676485 2.457907 0.276 |
| **Uterine volume at 6 months**  Side Direct Indirect Difference  Coef. Std. Err. Coef. Std. Err. Coef. Std. Err. P>\|z\|  A B -28.5 34.81713 -7.518966 42.33556 -20.98103 54.81362 0.702  A C 17 34.34081 -3.980595 42.72324 20.98059 54.81392 0.702  B C 24.51891 24.76042 45.5 48.90357 -20.98109 54.81457 0.702 |
| **Irregular uterine bleeding**  Side Direct Indirect Difference  Coef. Std. Err. Coef. Std. Err. Coef. Std. Err. P>\|z\|  A B 3.953805 1.003536 .9191943 385.1138 3.03461 385.115 0.994  B C -.0868146 .5562415 -7.886746 918.3932 7.799932 918.3934 0.993  B D -3.92729 1.243581 -6.410089 1347.164 2.482799 1347.166 0.999 |
| **Hot flashes**  Side Direct Indirect Difference  Coef. Std. Err. Coef. Std. Err. Coef. Std. Err. P>\|z\|  A B* . . . . . . .  A C* . . . . . . .  *No detectable source of inconsistency |
| **Breast tenderness**  Side Direct Indirect Difference  Coef. Std. Err. Coef. Std. Err. Coef. Std. Err. P>\|z\|  A B .7637647 .6497057 .0185367 876.1272 .745228 876.1275 0.999  B C -.045242 .5432398 -1.536477 1963.184 1.491235 1963.184 0.999 |

**Table S3.** SIDE analysis for study outcomes.
