# Supplementary material for: Efficacy and safety of hormone therapies for treating adenomyosis-associated pelvic pain: a systematic review and network meta-analysis of randomized controlled trials
Source: Front Endocrinol (Lausanne). 2025 Mar 17;16:1571727. doi: 10.3389/fendo.2025.1571727 (PMC11955467; doi:10.3389/fendo.2025.1571727)
Supplement: Supplementary file 6 [file Table4.docx]

|  | Irregular uterine bleeding | Hot flashes | Breast tenderness |
| --- | --- | --- | --- |
| Studies (n) | 4^25,26,28,29^ | 3^25,26,29^ | 3^26,28,29^ |
| Treatments | COC  DNG  LNG-IUS  Placebo | COC  DNG  LNG-IUS  Placebo | COC  DNG  LNG-IUS |
| SUCRA rank | COC (50.3%)  Placebo (49.7%)  LNG-IUS (0%)  DNG (0%) | COC (69.8%)  Placebo (18.6%)  LNG-IUS (11.5%)  DNG (0%) | COC (76.7%)  LNG-IUS (17.6%)  DNG (5.7%) |

Table S4. Common adverse effects for hormone treatment of adenomyosis.

COC: combined oral contraceptive; LNG-IUS: levonorgestrel Intrauterine system; DNG: dienogest; SUCRA: Surface Under the Cumulative Ranking curve Area.
